# Impact of mirabegron versus solifenacin on autonomic function and arterial stiffness in female overactive bladder syndrome: a randomized controlled trial

**DOI:** 10.1038/s41598-022-18391-6

**Published:** 2022-08-20

**Authors:** Sheng-Mou Hsiao, Fung-Chao Tu, Ta-Chen Su, Pei-Chi Wu, Ho-Hsiung Lin

**Affiliations:** 1 Graduate School of Biotechnology and Bioengineering, Yuan Ze University, Taoyuan, Taiwan; 2 Department of Obstetrics and Gynecology, Far Eastern Memorial Hospital, Banqiao, New Taipei, Taiwan; 3 Department of Obstetrics and Gynecology, National Taiwan University College of Medicine and Hospital, No. 8 Chung-Shan South Road, Taipei, 100 Taiwan; 4 Department of Internal Medicine, National Taiwan University College of Medicine and Hospital, Taipei, Taiwan

**Keywords:** Bladder, Urinary incontinence, Urological manifestations

## Abstract

The study aims to elucidate the impact of mirabegron versus solifenacin on autonomic function and peripheral arterial conditions in women with overactive bladder syndrome (OAB). All consecutive women with OAB were randomized to receive 12 weeks of mirabegron 25 mg or solifenacin 5 mg once per day. Heart rate variability, cardio-ankle vascular index, ankle-brachial pressure index, blood pressure, and heart rate were compared between the two groups. There were 87 women (mirabegron, n = 43; and solifenacin, n = 44) who completed 12-week treatment and underwent heart rate variability examination. Systolic blood pressure (median: − 4.5 to − 5.5 mmHg) and diastolic blood pressure (median: − 0.5 to − 3.5 mmHg) decreased after solifenacin treatment, and heart rate (median: + 2 bpm) increased after mirabegron treatment, despite of no between-group difference. In addition, posttreatment heart rate variability, cardio-ankle vascular index, and ankle-brachial pressure index did not differ compared with baseline; and there were no between-group differences. In conclusion, solifenacin might decrease blood pressure, and mirabegron might increase heart rate. Nonetheless, there were no significant impacts of 12-week mirabegron versus solifenacin treatment on autonomic function and arterial stiffness.

## Introduction

Overactive bladder syndrome (OAB) is characterized by the core symptom of urinary urgency, usually accompanied by frequency and nocturia^1^. Many theories, such as myogenic, neurogenic and autonomous theories, have been considered as possible etiologies of OAB^2^. Beta-3 agonists and antimuscarinics are the main medications for OAB.

Beta-1 and beta-2 adrenoceptors coexist in the human heart with beta-1 predominating with a ratio of approximately 70:30 in the atria and 80:20 in the ventricles^3^. Mirabegron is a beta-3 agonist; however, mirabegron may activate the beta-1 receptor^4^. Mo et al. found that mirabegron increases the force of atrial contraction through beta-1 receptor, but not beta-3 receptor^5^. Besides, beta-3 receptor was found in the vascular system^4^, and beta-3 agonists may affect the peripheral vascular system.

Antimuscarinics, including solifenacin and tolterodine, remain the mainstay drugs for OAB. Solifenacin has a moderate selectivity for the M3 receptor; however, solifenacin could stimulate M2 receptors^6^. Stimulation of cardiac M2 receptors modulates pacemaker activity and atrioventricular conduction and cardiac contraction^7^. In addition, the M3 receptor is associated with endothelium-dependent, acetylcholine-induced vasodilation^8^. In the thoracic aortic rings of M3 receptor knockout mice, the vasodilatory effect of the cholinergic agonists was greatly decreased^8^. Thus, the issue of whether solifenacin may affect heart and peripheral vascular system may be worth studying.

Heart rate variability is frequently used to assess autonomic dysfunction. Higher cardiovascular risk was found in patients with autonomic dysfunction^9^. In addition, OAB women were found to have higher severity of autonomic dysfunction, compared with normal controls^9,10^. Some antimuscarinics (e.g., tolterodine and propiverine) were found to be detrimental to autonomic function^9,11^. In addition, arterial stiffness and lower extremity atherosclerosis, which were associated with higher cardiac risk, have been used as a surrogate for peripheral arterial conditions^12,13^.

It has been reported that antimuscarinics might improve arterial stiffness^9^. However, there was no report mentioning about the impact of mirabegron on the peripheral arterial conditions. Thus, we were interested whether mirabegron can affect the peripheral arterial conditions.

Combination treatment (i.e., mirabegron plus solifenacin) has been found to have an additive effect, compared with monotherapy^14,15^; and the side effect of solifenacin is different to mirabegron^16^. Thus, probably related to side effects and cost considerations, monotherapy is frequently used as an initial treatment for women with OAB^17^. We were interested about the impact of mirabegron versus solifenacin monotherapy on autonomic function and peripheral arterial conditions. Thus, the main objective of this study was to investigate the impact of mirabegron versus solifenacin on autonomic function and peripheral arterial conditions (such as arterial stiffness and narrowing of the lower extremites arteries).

## Results

**Figure 1:**
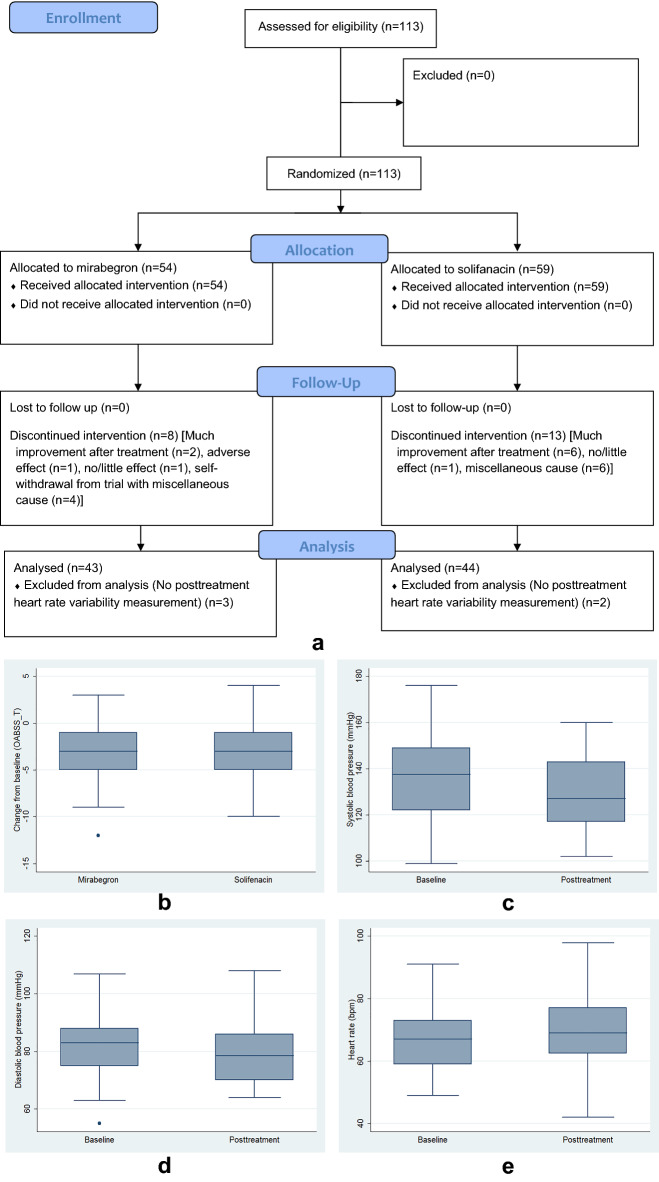
(**a**) The CONSORT 21010 flow diagram of participants with overactive bladder syndrome. (**b**) Box plots of the posttreatment change from baseline of the total score of OABSS (OABSS_T) between the mirabegron and solifenacin groups. Box plots of (**c**) systolic blood pressure and (**d**) diastolic blood pressure measured from right brachial artery at baseline and 12 weeks after solifenacin treatment. (**e**) Box plots of heart rate at baseline and 12 weeks after mirabegron treatment.

Between September 2015 and May 2020, a total of 113 women were enrolled in this study. However, 21 women discontinued treatment during the 12-week treatment period and were excluded from the final analysis (Fig. 1a).

There were 92 women (mirabegron, n = 46; and solifenacin, n = 46) who completed 12-week treatment. However, 5 patients did not receive heart rate variability examinations; thus, a total of 87 women (mirabegron, n = 43; and solifenacin, n = 44) were analyzed (Fig. 1a). Except nocturia, urgency incontinence, systolic blood pressure and diastolic blood pressure, there were no between-group differences (Table 1).

**Table 1:** Comparison of baseline data of overactive bladder symptoms, heart rate variability, cardio-ankle vascular index and ankle-brachial pressure index between the mirabegron and solifenacin groups.

Variables	Mirabegron (n = 43)	Solifenacin (n = 44)	*P*†
Age (years)	59 (48, 65)	63 (53, 67)	0.24
Body mass index (kg/m^2^)	23.2 (21.4, 27.0)	24.2 (21.8, 25.3)	0.89
**OABSS**			
Daytime frequency	1 (1, 1)	1 (1, 1)	0.43
Nocturia	2 (2, 3)	2 (1, 3)	0.04
Urgency	4 (3, 4)	4 (3, 4)	0.67
Urgency incontinence	1 (0, 3)	2 (1, 4)	0.005
OABSS total score	8 (7, 9)	9 (8, 11)	0.23
SDNN (ms)	27.4 (20.8, 45.9)	27.8 (17.2, 38.5)	0.79
RMSSD (ms)	24.0 (14.0, 36.0)	20.3 (14.4, 33.0)	0.78
NN50 count	5.0 (0.0, 28.0)	3.5 (0.1, 13)	0.90
pNN50 (%)	1.6 (0.0, 10.7)	1.0 (0.0, 4.5)	0.81
SD1	16.8 (9.9, 25.5)	14.4 (10.2, 23.3)	0.76
SD2	32.6 (24.5)	35.1 (21.3, 48.5)	0.79
SD1/SD2	0.47 (0.33, 0.64)	0.45 (0.36, 0.59)	0.86
VLF (ms^2^)	162.5 (74.5, 300)	204.8 (72.0, 316.6)	0.71
LF (ms^2^)	81.0 (30.0, 225.0)	56.3 (23.6, 157.1)	0.20
HF (ms^2^)	109.5 (28.5, 286.5)	75.3 (27.3, 174.1)	0.43
Total power (ms^2^)	355.5 (189.0, 983.5)	357.5 (137.6, 701.0)	0.48
LF norm (n.u.)	43.6 (38.0, 62.2)	42.7 (30.9, 58.2)	0.23
HF norm (n.u.)	56.3 (37.8, 62.0)	57.3 (41.8, 68.9)	0.23
LF/HF	0.83 (0.64, 1.70)	0.80 (0.48, 1.40)	0.28
SBP of right brachial artery (mmHg)	125 (115, 137)	138 (122, 149)	0.01
DBP of right brachial artery (mmHg)	78 (72, 83)	82.5 (75, 88)	0.04
SBP of left brachial artery (mmHg)	125 (113, 136)	136 (123, 144.8)	0.006
DBP of left brachial artery (mmHg)	78 (72, 84)	82.5 (75, 88)	0.04
SBP of right ankle artery (mmHg)	139 ( 121, 153)	149 (133, 163.8)	0.02
DBP of right ankle artery (mmHg)	74 (70, 80)	78 (70.5, 84)	0.052
SBP of left ankle artery (mmHg)	136 (125, 153)	153 (137.5, 161.8)	0.01
DBP of left ankle artery (mmHg)	74 (69, 80)	79 (71.5, 84)	0.051
Hear rate (per min)	67 (58, 73)	71 (62.3, 77)	0.13
CAVI	7.8 (7.1, 8.9)	8.5 (7.7, 9.1)	0.15
ABI	1.09 (1.05, 1.11)	1.08 (1.04, 1.12)	0.91

Data are expressed as median (interquartile range).

*AA* ankle artery, *ABI* ankle-brachial pressure index, *BA* brachial artery, *CAVI* cardio-ankle vascular index, *DBP* diastolic blood pressure, *HF* power in high frequency, *HF norm* HF power in normalized units, *LF* power in low frequency, *LF norm* LF power in normalized units, *LF/HF* the ratio of LF/HF, *NN50 count* number of pairs of adjacent normal-to-normal (NN) intervals differing by more than 50 ms in the entire recording, *OABSS* Overactive Bladder Symptoms Score, *pNN50* NN50 count divided by the total number of all normal-to-normal interval, *RMSSD* the square root of the mean squared successive differences of normal to normal intervals, *SBP* systolic blood pressure, *SD1* standard deviation of instantaneous beat-to-beat RR interval variability, *SD2* standard deviation of continuous beat-to-beat RR interval variability, *SD1/SD2* the ratio of SD1/SD2, *SDNN* standard deviation of NN RR intervals in electrocardiography, *USS* Urgency Severity Scale, *VLF* power in low frequency range.

^†^By Wilcoxon rank-sum test.

The Overactive Bladder Symptom Score (OABSS) subscores and total score were improved after treatment (Fig. 1b); however, there were no between-group differences in the changes from baseline after 12-week treatment (Table 2).

**Table 2:** Comparison of the changes from baseline in overactive bladder symptoms, heart rate variability cardio-ankle vascular index and ankle-brachial pressure index after 12-week treatment between the mirabegron and solifenacin groups.

Variable	Mirabegron (n = 43)^†^	Solifenacin (n = 44)^†^	*P*^‡^
**OABSS**			
Daytime frequency	0 (− 1, 0)**	0 (− 1, 0)**	0.23
Nocturia	0 (− 1, 0)**	0 (− 1, 0)*	0.17
Urgency	− 1 (− 3, 0)**	− 1 (− 3, 0)**	0.71
Urgency incontinence	0 (− 1, 0)*	− 1 (− 2, 0)**	0.11
OABSS total score	− 3 (− 5, − 1)**	− 3 (− 5, − 1)**	0.75
SDNN (ms)	− 1.9 (− 11.4, 5.4)	1.2 (− 5.4, 6.1)	0.14
RMSSD (ms)	− 2.1 (− 12.5, 6.5)	0.0 (− 5.5, 9.0)	0.29
NN50	0 (− 5.5, 2.5)	0 (− 5.1, 4.4)	0.81
pNN50 (%)	0 (− 2.2, 0.8)	0 (− 0.7, 2.5)	0.26
SD1	− 1.5 (− 8.8, 4.6)	0.0 (− 3.9, 6.5)	0.27
SD2	− 5.3 (− 17.8, 5.7)	0.8 (− 7.7, 8.6)	0.14
SD1/SD2	− 0.01 (− 0.14, 0.14)	0.01 (− 0.06, 0.14)	0.44
VLF(ms^2^)	− 53 (− 138.5, 81)	15.8 (− 72.3, 67.8)	0.12
LF (ms^2^)	− 2 (− 96.5, 41)	− 0.8 (− 22.4, 89.8)	0.33
HF (ms^2^)	− 3 (− 125, 52.5)	2.8 (− 51.9, 200.8)	0.23
Total Power (ms^2^)	− 35.5 (− 432, 185.5)	6.3 (− 146.1, 509.5)	0.20
LF norm (n.u.)	0.4 (− 12.3, 8.7)	− 3.0 (− 10.5, 9.0)	0.71
HF norm (n.u.)	− 0.4 (− 8.7, 12.3)	3.0 (− 9.0, 10.6)	0.70
LF/HF	0.02 (− 0.48, 0.25)	− 0.13 (− 0.45, 0.25)	0.89
SBP of right brachial artery (mmHg)	0 (− 12, 7)	− 5.5 (− 14.8, 1.3)**	0.09
DBP of right brachial artery (mmHg)	− 0.5 (− 5.3, 6)	− 3 (− 7, 3)*	0.24
SBP of left brachial artery (mmHg)	0 (− 6.3, 8.3)	− 4.5 (− 13.8, 5)*	0.13
DBP of left brachial artery (mmHg)	− 2 (− 7.3, 3)	− 3.5 (− 10.8, 2)**	0.25
SBP of right ankle artery (mmHg)	0 (− 10.5, 7.3)	− 4.5 (− 10.8, 4.8)*	0.33
DBP of right ankle artery (mmHg)	0.5 (− 4, 3)	− 0.5 (− 8.8, 4.8)	0.71
SBP of left ankle artery (mmHg)	− 2 (− 9, 4.8)	− 5.5 (− 16.8, 4.8)*	0.21
DBP of left ankle artery (mmHg)	− 1 (− 5, 2)	− 1 (− 7.8, 4.5)	0.17
Heart rate (per min)	2 (− 2.3, 7)*	1 (− 4, 7)	0.49
CAVI	− 0.05 (− 0.44, 0.40)	− 0.05 (− 0.69, 0.66)	0.88
ABI	0.01 (− 0.05, 0.05)	0.00 (− 0.04, 0.05)	0.99

Data are expressed as median (interquartile range). Changes from baseline are the subtraction of pretreatment values from posttreatment values. Abbreviations are as in Table 1.

^†^Within-group comparisons of baseline and posttreatment values were performed by Wilcoxon signed-rank test. *p < 0.05, and **p < 0.01.

^‡^Wilcoxon rank-sum test.

After 12-week treatment, all parameters of heart rate variability, including the standard deviation of the normal-to-normal (NN) intervals (SDNN), the square root of the mean squared differences of successive NN intervals (RMSSD), the number of pairs of adjacent normal-to-normal (NN) intervals differing by more than 50 ms in the entire recording (NN50 count), NN50 count divided by the total number of all normal-to-normal interval (pNN50), standard deviation of instantaneous beat-to-beat RR interval variability in the Poincaré Plot (SD1), standard deviation of continuous beat-to-beat RR interval variability in the Poincaré Plot (SD2), SD1/SD2 ratio, very low frequency (VLF), low frequency (LF), high frequency (HF), LF/HF ratio and total power did not differ compared with baseline; and there were no between-group differences in the changes from baseline (Table 2).

Posttreatment cardio-ankle vascular index (CAVI) and ankle-brachial pressure index (ABI) data did not differ compared with baseline; and there were no between-group differences in the changes from baseline (Table 2). However, systolic blood pressures, which were measured from the right brachial artery (Fig. 1c), left brachial artery, right ankle artery, and left ankle artery, were significantly decreased after solifenacin treatment, despite of no between-group differences (Table 2). Similarly, diastolic blood pressures, which were measured from the right brachial artery (Fig. 1d) and left brachial artery, were also decreased after solifenacin treatment, despite of no between-group differences (Table 2). In addition, the heart rate was increased after mirabegron treatment (Fig. 1e), despite of no between-group differences (Table 2).

## Discussion

In this study, there was no within- and between-group difference in heart rate variability after solifenacin versus mirabegron treatment (Table 2). Heart rate variability is used as a surrogate of autonomic function. Women with OAB were reported to have a higher severity of autonomic dysfunction, and tolterodine might deteriorate autonomic dysfunction^9,18^, but not solifenacin^9^. To our knowledge, there was no literature mentioned about the impact of mirabegron on heart rate variability. Taking together with the findings of a prior study (i.e., lack of effect of solifenacin on heart rate variability)^9^ and the current study (i.e., lack of effect of solifenacin and mirabegron on heart rate variability, Table 2), mirabegron seems to have no significant impact on autonomic function.

After solifenacin or mirabegron treatment, posttreatment CAVI did not differ compared with baseline. Similarly, it has been reported that CAVI did not improve after solifenacin treatment^9^. Taking together with our current study, CAVI did not change after solifenacin or mirabegron treatment (Table 2). It seems reasonable that CAVI did not improve after mirabegron treatment. CAVI is used to measure arterial stiffness; thus, mirabegron seems to have no impact on arterial stiffness.

ABI did not change after solifenacin or mirabegron treatment in this study. Similarly, it has been reported that tolterodine and solifenacin did not have an impact on ABI^9^. ABI is a measure of the narrowing of lower extremity arteries, such as atherosclerosis. Based on our findings of no significant change of ABI after 12-week solifenacin or mirabegron treatment (Table 2), mirabegron seems to have no short-term impact on the narrowing of lower extremity arteries. Nonetheless, atherosclerosis takes years to develop. Despite the ABI did not change from baseline after 12-week treatment, our findings cannot be extrapolated to the conclusion of no long-term impact on lower extremity atherosclerosis.

Systolic blood pressure and diastolic blood pressure decreased after solifenacin treatment (Fig. 1c,d), but not mirabegron (Table 2). Similarly, Drake et al. found a decrease of systolic blood pressure (mean change from baseline: − 1.41 mmHg) and diastolic blood pressure (mean change from baseline: − 0.76 mmHg) after solifenacin treatment^19^. However, the magnitudes of change from baseline in systolic blood pressure (change from baseline of the left brachial artery: − 4.1 ± 13.6 mmHg) and diastolic blood pressure (change from baseline of the left brachial artery: − 4.5 ± 8.2 mmHg) seems to be larger in our study. Moreover, mirabegron could increase blood pressure (i.e., an increase of 0.4–0.6 mmHg after 12-week 50 mg mirabegron)^20^, and mirabegron is contraindicated to treat OAB patients with severe uncontrolled high blood pressure. Contrarily, a decrease of blood pressure was found in our OAB patients receiving solifenacin treatment. Thus, our findings justify that solifenacin is a good alternative for OAB women with hypertension.

Heart rate increased after mirabegron treatment (mean change from baseline: 0.9 ± 9.2 bpm, Fig. 1e), but not solifenacin. Similarly, Nitti et al. found an increase in heart rate after mirabegron treatment (adjusted change from baseline: 1.3 bpm in the morning measurement and 0.2 bpm in the afternoon measurement)^21^. The increase in heart rate is probably mediated by cross-activation of the β1-adrenoceptor^4^, as mirabegron has a greater affinity to the canine β1-receptor^22^. Therefore, mirabegron may be unsuitable for women whose resting heart rate is of concern.

In this study, OABSS subscores and total score improved after mirabegron and solifenacin treatments, but no between-group differences (Table 2). Similarly, a pooled data from 10 randomized controlled trials revealed a similar therapeutic efficacy between mirabegron and solifenacin^23^.

There were some limitations in this study. The sample size was limited, and the enrolled time interval was long. In addition, mirabegron 25 mg, but not mirabegron 50 mg, was used in this study. Furthermore, most OAB women in this study were neurologically intact. Thus, our results might not be extrapolated to those women with neurological deficiency.

In conclusion, solifenacin might decrease blood pressure, and mirabegron might increase heart rate. Nonetheless, there were no significant impacts of 12-week mirabegron versus solifenacin treatment on autonomic function and arterial stiffness.

## Methods

This study was conducted at the Department of Obstetrics and Gynecology at a tertiary referral center. National Taiwan University Hospital Research Ethics Committee approved the study protocol (No. 20156092MIND). The study design was prospective, randomized, controlled, and open labeled. This study has been registered with ClinicalTrials.gov (NCT02540707, date of registration: 04/09/2015).

The screening visit was designed as visit 0, and the inclusion criteria included: (1) women aged at least 20 years who had at least a 3-month history of OAB symptoms, including urgency, urinary frequency, nocturia or urgency incontinence; (2) an average of ≥ 8 micturitions per 24 h period^6^. Exclusion criteria included clinically significant dysuria, severe stress urinary incontinence or mixed urinary incontinence with dominant stress incontinence, and other symptoms that were contraindications for antimuscarinic medication or beta-3 agonists^6^.

Urodynamic studies and a 20-min pad test^24–26^ were also performed between visit 0 and visit 1. Eligibility was determined at visit 1 (baseline, one week after visit 0) using the results recorded in the 3-day bladder diary prior to visit 1. Patients were randomized into two groups using a computer-generated random number list to receive either mirabegron 25 mg or solifenacin 5 mg once a day for 12 weeks. The randomization sequence was created using Excel 2007 (Microsoft, Redmond, WA, USA) with a 1:1 allocation using simple randomization. Patients were followed up at week 4 (visit 2), week 8 (visit 3), and week 12 (visit 4).

The enrolled participants were requested to complete the following questionnaires, urodynamic studies, and the following examinations before and after 12 weeks’ treatment, including OABSS^27^, standard 12-lead electrocardiography, 15 min of Holter monitoring, CAVI and ABI.

### Heart rate variability measurements

Heart rate variability was measured using a continuous ambulatory Holter electrocardiographic recorder (model 3100 A, Philips Medical System, Andover, Massachusetts) with a sampling rate of 250 Hz (4 ms after emptying her bladder and resting for 30 min**.** The electrocardiographic signals were recorded for 15 min. The QRS complexes were automatically classified and manually verified as normal sinus rhythm, atrial or ventricular premature beats, or noise by comparison with adjacent QRS morphology. The R–R intervals were deduced from the adjacent normal sinus beats, and their interval time series were processed by a program written in the Matlab language (version 5.2, Mathworks Inc., Natick, MA, USA)^9,28^.

The time domain parameters, including SDNN, RMSSD, NN50 count, pNN50, SD1, SD2, and the ratio of SD1/SD2 were obtained. SDNN is an estimate of overall heart rate variability; and RMSSD is an estimate of high-frequency variations in heart rate^9,29^.

The frequent domain parameters, including total power, VLF, LF, HF, LF power in normalized units (LF norm), HF power in normalized units (HF norm), and the LF/HF ratio were also obtained. The power spectral density was calculated by the nonparametric method (i.e., the fast Fourier transform algorithm). The efferent vagal activity is a major contributor to the HF component. LF component is considered as a marker of sympathetic modulation or both sympathetic and vagal influences^9,29^.

### CAVI and ABI measurements

Arterial stiffness was measured by the CAVI with the VaSera VS-1000 vascular screening system (FuKuda Denshi Co., Ltd., Tokyo, Japan)^30,31^. CAVI is a new index of arterial stiffness and independent of blood pressure, which is compatible with conventional aortic pulse wave velocity. The procedure was performed as follows. After the participants sitting and resting for at least five minutes, the measurements were taken with the patients in the supine position, with monitoring cuffs attached to the right or left upper arm and ankle to detect brachial and ankle pulse waves. Heart sounds and electrocardiograms were also monitored. The pulse wave velocity from the heart to the ankle was calculated by measuring length from the aortic value to the ankle and dividing by time, which was determined according to the heart sound and the rise of the brachial and ankle pulse waves. Blood pressure was also measured at the brachial artery^32^. Thus, the CAVI can be calculated by the equation: a[(2ρ/∆P) × ln(Ps/Pd)PWV2] + b. (a and b: constants; PWV, cardio-ankle pulse wave velocity; ∆P, Ps-Pd; ln, natural logarithm; Pd, diastolic blood pressure; Ps, systolic blood pressure; ρ, blood density)^30,31^, and the average of the left and right CAVI values was used for analysis^9^.

The ABI is a noninvasive method to check peripheral artery conditions and an indicator of lower extremity atherosclerosis. The ABI was measured using the above device with cuffs that can simultaneously measure blood pressure levels in both arms (i.e., brachial artery) and both legs (i.e., ankle artery). The ABI was calculated separately for each leg, and the lower of the two ABI values was used for analysis^9,33^. A low ABI may indicate narrowing of the lower extremity arteries.

All terminology used in this paper conforms to the standards recommended by the International Urogynecological Association and International Continence Society joint report^1^. The flow diagram was completed according to the CONSORT 2010 standard. Stata software (Version 11.0; StataCorp, College Station, Texas, USA) was used for statistical analyses. The Wilcoxon rank-sum test and Wilcoxon signed-rank test were used as statistical methods, as appropriate. A P value of less than 0.05 was considered statistically significant. The primary objective of this study was to compare the impact of mirabegron versus solifenacin on heart rate variability. Secondary objectives of this study included comparison of the CAVI, ABI, heart rate, and blood pressure between the mirabegron and solifenacin groups. Based on a previous study about the comparison of the impact of solifenacin versus tolterodine on heart rate variability^9^, we concluded that about 43 subjects in each group were required to test the null hypothesis.

### Research involving human participants and/or animals

This study was performed in line with the principles of the Declaration of Helsinki. Approval was granted by the Research Ethics Committee of National Taiwan University Hospital (Ethics approval number: 20156092MIND).

### Informed consent

Informed consent was obtained from all individual participants included in the study.

## Data Availability

The datasets generated during and/or analyzed during the current study are available from the corresponding author on reasonable request.

## References

[1] Haylen BT (2010). An International Urogynecological Association (IUGA)/International Continence Society (ICS) joint report on the terminology for female pelvic floor dysfunction. Int. Urogynecol. J..

[2] Drake MJ, Mills IW, Gillespie JI (2001). Model of peripheral autonomous modules and a myovesical plexus in normal and overactive bladder function. Lancet.

[3] Brodde OE, Bruck H, Leineweber K (2006). Cardiac adrenoceptors: Physiological and pathophysiological relevance. J. Pharmacol. Sci..

[4] Rosa GM (2018). Cardiovascular effects of antimuscarinic agents and beta 3-adrenergic receptor agonist for the treatment of overactive bladder. Expert Opin. Drug. Saf..

[5] Mo W, Michel MC, Lee XW, Kaumann AJ, Molenaar P (2017). The β3-adrenoceptor agonist mirabegron increases human atrial force through β1-adrenoceptors: An indirect mechanism?. Br. J. Pharmacol..

[6] Hsiao SM, Chang TC, Lin HH (2019). The probability of re-treatment after discontinuation of a 3-month versus a 6-month course of solifenacin for female overactive bladder: A prospective randomized controlled study. Maturitas.

[7] Andersson KE, Campeau L, Olshansky B (2011). Cardiac effects of muscarinic receptor antagonists used for voiding dysfunction. Br. J. Clin. Pharmacol..

[8] Khurana S (2004). Vasodilatory effects of cholinergic agonists are greatly diminished in aorta from M3R−/− mice. Eur. J. Pharmacol..

[9] Hsiao SM, Su TC, Chen CH, Chang TC, Lin HH (2014). Autonomic dysfunction and arterial stiffness in female overactive bladder patients and antimuscarinics related effects. Maturitas.

[10] Choi JB, Kim YB, Kim BT, Kim YS (2005). Analysis of heart rate variability in female patients with overactive bladder. Urology.

[11] Abrams P (2006). Comparison of the efficacy, safety, and tolerability of propiverine and oxybutynin for the treatment of overactive bladder syndrome. Int. J. Urol..

[12] Maldonado J (2011). Arterial stiffness predicts cardiovascular outcome in a low-to-moderate cardiovascular risk population: The EDIVA (Estudo de DIstensibilidade VAscular) project. J. Hypertens..

[13] Murabito JM (2003). The ankle-brachial index in the elderly and risk of stroke, coronary disease, and death: the Framingham Study. Arch. Intern. Med..

[14] Herschorn S (2017). Efficacy and safety of combinations of mirabegron and solifenacin compared with monotherapy and placebo in patients with overactive bladder (SYNERGY study). BJU Int..

[15] Yamaguchi O (2015). Safety and efficacy of mirabegron as 'add-on' therapy in patients with overactive bladder treated with solifenacin: A post-marketing, open-label study in Japan (MILAI study). BJU Int..

[16] Vecchioli Scaldazza C, Morosetti C (2016). Comparison of therapeutic efficacy and urodynamic findings of solifenacin succinate versus mirabegron in women with overactive bladder syndrome: Results of a randomized controlled study. Urol. Int..

[17] Wang J (2019). Meta-analysis of the efficacy and safety of mirabegron and solifenacin monotherapy for overactive bladder. Neurourol. Urodyn..

[18] Schiffers M, Sauermann P, Schurch B, Mehnert U (2010). The effect of tolterodine 4 and 8 mg on the heart rate variability in healthy subjects. World J. Urol..

[19] Drake MJ (2017). Cardiovascular safety in refractory incontinent patients with overactive bladder receiving add-on mirabegron therapy to solifenacin (BESIDE). Int. J. Clin. Pract..

[20] Nitti VW (2014). Safety and tolerability of the β3 -adrenoceptor agonist mirabegron, for the treatment of overactive bladder: Results of a prospective pooled analysis of three 12-week randomised phase III trials and of a 1-year randomised Phase III trial. Int. J. Clin. Pract..

[21] Nitti VW (2013). Mirabegron for the treatment of overactive bladder: A prespecified pooled efficacy analysis and pooled safety analysis of three randomised, double-blind, placebo-controlled, phase III studies. Int. J. Clin. Pract..

[22] Shen YT, Cervoni P, Claus T, Vatner SF (1996). Differences in β3-adrenergic receptor cardiovascular regulation in conscious primates, rats and dogs. J. Pharmacol. Exp. Ther..

[23] Chapple CR (2020). Safety and efficacy of mirabegron: Analysis of a large integrated clinical trial database of patients with overactive bladder receiving mirabegron, antimuscarinics, or placebo. Eur. Urol..

[24] Sand PK (1992). The evaluation of the incontinence female. Curr. Probl. Obstet. Gynecol. Fertil..

[25] Wu WY, Sheu BC, Lin HH (2006). Comparison of 20-minute pad test versus 1-hour pad test in women with stress urinary incontinence. Urology.

[26] Wu WY, Hsiao SM, Wu PC, Lin HH (2020). Test–retest reliability of the 20-min pad test with infusion of strong-desired volume in the bladder for female urodynamic stress incontinence. Sci. Rep..

[27] Homma Y (2006). Symptom assessment tool for overactive bladder syndrome–overactive bladder symptom score. Urology.

[28] Su TC (2008). Elevated blood pressure, decreased heart rate variability and incomplete blood pressure recovery after a 12-hour night shift work. J. Occup. Health..

[29] Task Force of the European Society of Cardiology and the North American Society of Pacing and Electrophysiology. Heart rate variability: Standards of measurement, physiological interpretation and clinical use. *Circulation* **93**, 1043–1065 (1996). 8598068

[30] Shirai K, Utino J, Otsuka K, Takata M (2006). A novel blood pressure-independent arterial wall stiffness parameter; cardio-ankle vascular index (CAVI). J. Atheroscler. Thromb..

[31] Shirai K (2011). Cardio-ankle vascular index (CAVI) as a novel indicator of arterial stiffness: Theory, evidence and perspectives. J. Atheroscler. Thromb..

[32] Yukutake T (2014). Arterial stiffness determined according to the cardio-ankle vascular index (CAVI) is associated with mild cognitive decline in community-dwelling elderly subjects. J. Atheroscler. Thromb..

[33] Huang CL (2007). Postchallenge hyperglycaemic spike associate with arterial stiffness. Int. J. Clin. Pract..

